# Crystal structure of potassium hydrogen bis­((*E*)-2-{4-[3-(thio­phen-3-yl)acrylo­yl]phen­oxy}acetate)

**DOI:** 10.1107/S2056989021004801

**Published:** 2021-05-11

**Authors:** Trung Vu Quoc, Linh Phan Thuy, Dai Do Ba, Duong Tran Thi Thuy, Linh Nguyen Ngoc, Chinh Nguyen Thuy, Linh Duong Khanh, Hung Ha Manh, Hoang Thai, Khoe Le Van, Luc Van Meervelt

**Affiliations:** a Faculty of Chemistry, Hanoi National University of Education, 136 Xuan Thuy, Cau Giay, Hanoi 10000, Vietnam; b High School for Gifted Students, Hanoi National University of Education, 136 Xuan Thuy, Cau Giay, Hanoi 10000, Vietnam; c Nguyen Trai High School, 50 Nam Cao Street, Ba Dinh, Hanoi 10000, Vietnam; d Bien Hoa Gifted High School, 86 Chu Van An Street, Phu Ly City, Ha Nam Province, Vietnam; e Faculty of Training Bachelor of Practice, Than Do University, Kim Chung, Hoai Duc, Hanoi 10000, Vietnam; f Institute of Tropical Technology, Vietnam Academy of Science and Technology, 18 Hoang Quoc Viet, Cau Giay, Hanoi 10000, Vietnam; g Graduate University of Science and Technology, Vietnam Academy of Science and Technology, 18 Hoang Quoc Viet, Cau Giay, Hanoi 10000, Vietnam; h Faculty of General Education, Hanoi University of Mining and Geology, Duc Thang Ward, Bac Tu Liem District, Hanoi 10000, Vietnam; i Faculty of Natural Sciences, Hong Duc University, 565 Quang Trung, Dong Ve Ward, Thanh Hoa City, Vietnam; j Department of Chemistry, KU Leuven, Biomolecular Architecture, Celestijnenlaan 200F, Leuven (Heverlee), B-3001, Belgium

**Keywords:** crystal structure, hydrogen bonding, potassium salt, thio­phene, Hirshfeld analysis

## Abstract

The synthesis and spectroscopic data of (*E*)-2-{4-[3-(thio­phen-3-yl)acrylo­yl]phen­oxy}acetic acid are described. Crystallization from an ethanol–water mixture resulted in the title compound, C_30_H_23_KO_8_S_2_ or [K(C_15_H_11_O_4_S)(C_15_H_12_O_4_S)]_*n*_, containing one mol­ecule of the acid and one mol­ecule of the potassium salt in the asymmetric unit. Both mol­ecules share the H atom between their carboxyl groups and a potassium ion. The C=C bonds display an *E* configuration. The thio­phene and phenyl rings in the two mol­ecules are inclined by 43.3 (2) and 22.7 (2)°. The potassium ion is octa­hedrally coordinated by six O atoms. This distorted octa­hedron shares on opposite sides two oxygen atoms with inversion-related octa­hedra, resulting in chains of octa­hedra running in the [010] direction, which form ladder-like chains by C—H⋯π inter­actions. A Hirshfeld surface analysis indicates that the highest contributions to the surface contacts arise from inter­actions in which H atoms are involved, with the most important contribution being from H⋯H (31.6 and 31.9% for the two mol­ecules) inter­actions.

## Abstract 2

The title compound, C_30_H_23_KO_8_S_2_, contains one mol­ecule of (*E*)-2-{4-[3-(thio­phen-3-yl)acrylo­yl]phen­oxy}acetic acid and one mol­ecule of its potassium salt in the asymmetric unit. The distorted KO_6_ octa­hedra share edges, resulting in chains running in the [010] direction.

## Chemical context   

Over the last two decades, water-soluble polythio­phenes and their derivatives have been of particular importance among conjugated polyelectrolytes owing to a unique combination of high conductivity, environmental stability and structural versatility, allowing derivatization of the π-conjugated backbone in view of numerous technological applications (Wang *et al.*, 2015 ▸; Chayer *et al.*, 1997 ▸; Wang *et al.*, 2013 ▸). Many regioregular polythio­phenes with pendant carb­oxy­lic acid functionality have been studied (Ewbank *et al.*, 2004 ▸; McCullough *et al.*, 1997 ▸; Wu *et al.*, 2015 ▸; Janáky *et al.*, 2010 ▸). The increased alkyl side-chain length allows for increased coplanarity of the main-chain thio­phene rings to advance regioregular polythio­phene backbones (Vu Quoc *et al.*, 2019*a* ▸). A lot of synthetic research has been conducted with a view to increasing the side-chain length of thio­phene rings (Vu Quoc *et al.*, 2020 ▸). Crystal studies of thio­phene monomers have also been reported (Vu Quoc *et al.*, 2017 ▸, 2018 ▸, 2019*b* ▸).
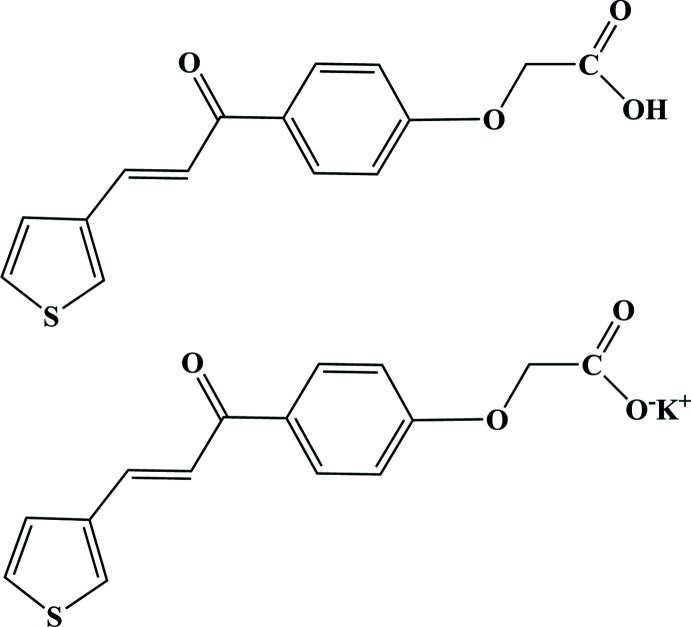


(*E*)-2-{4-[3-(thio­phen-3-yl)acrylo­yl]phen­oxy}acetic acid are reported. This compound is considered to be a good monomer for the synthesis of water-soluble polythio­phene-based conjugated polyelectrolytes. A single-crystal structure determination indicates that after crystallization, crystals were obtained containing one mol­ecule of the acid and one mol­ecule of the potassium salt in the asymmetric unit.

## Structural commentary   

**Figure 1:**
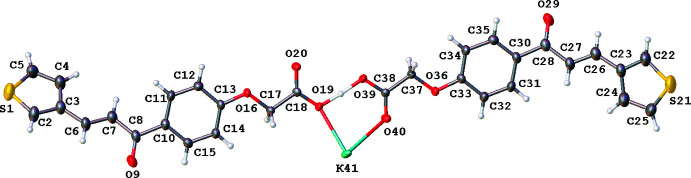
The mol­ecular structure of the title compound, showing the atom-labelling scheme and displacement ellipsoids at the 30% probability level.

The title compound crystallizes in the triclinic space group *P* 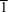 as a complex formed between the acid and the potassium salt of the acid, as illustrated in Fig. 1 ▸. In the following discussion, mol­ecule *A* includes atoms S1–O20 and mol­ecule *B* atoms S21–O40. Both mol­ecules share hydrogen atom H19 between their carboxyl groups and a potassium ion, K41. Atom H19 is involved in hydrogen-bonding inter­actions with atoms O39 and O40 (Table 1 ▸). The dihedral angle between the thio­phene and phenyl rings is 43.3 (2)° for mol­ecule *A* and 22.7 (2)° for mol­ecule *B*. The C=C bonds display an *E* configuration, resulting in short intra­molecular C6—H6⋯O9 and C26—H26⋯O29 inter­actions (Table 1 ▸). The terminal thio­phene groups are involved in intense thermal motion.

**Figure 2:**
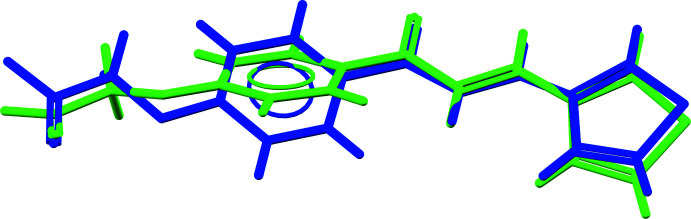
Overlay diagram of the two mol­ecules *A* (green) and *B* (blue), comprising the asymmetric unit. H atoms are hidden for clarity (*Mercury*; Macrae *et al.*, 2020 ▸).

Fig. 2 ▸ shows an overlay diagram of the two mol­ecules *A* and *B* [r.m.s. deviation 0.5622 Å as calculated using *Mercury* (Macrae *et al.*, 2020 ▸)]. The largest differences are caused by the different orientation of the phenyl groups.

**Figure 3:**
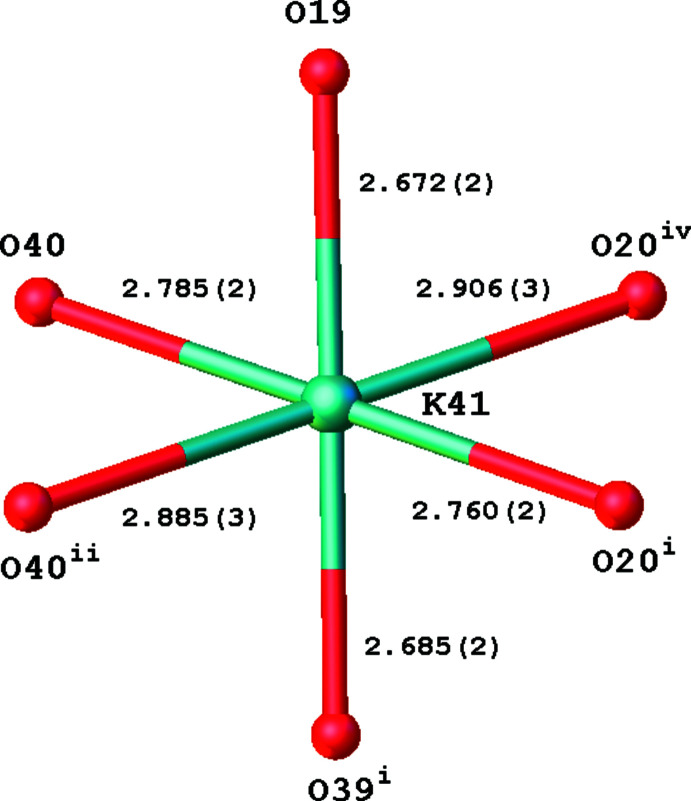
Octa­hedral coordination around K in the title compound [symmetry codes: (i) *x* + 1, *y*, *z*, (ii) −*x* + 2, −*y* + 2, −*z* + 1, (iv) −*x* + 1, −*y* + 1, −*z* + 1].

Potassium ion K41 is octa­hedrally coordinated by six O atoms with K—O distances between 2.672 (2) and 2.906 (3) Å (Fig. 3 ▸) and an octa­hedral volume of 21.871 Å^3^. The coord­ination sphere can be extended with atoms O16 and O36, but K⋯O distances are much longer [K41⋯O16^iv^ = 3.245 (3), K41⋯O36^ii^ = 3.347 (3) Å, symmetry codes: (ii) −*x* + 2, −*y* + 2, −*z* + 1, (iv) −*x* + 1, −*y* + 1, −*z* + 1].

## Supra­molecular features   

**Figure 4:**
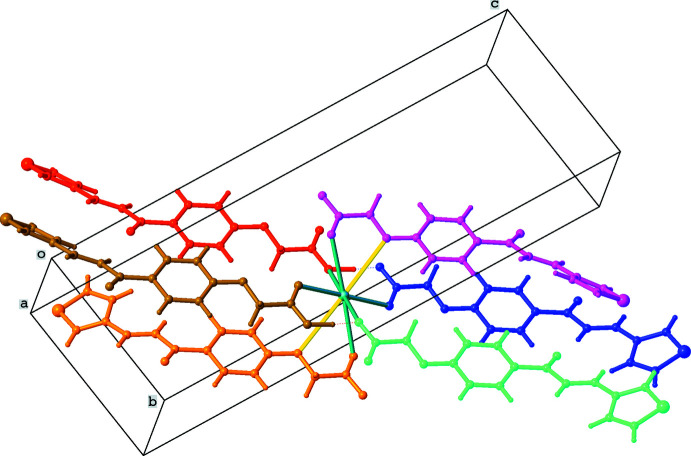
Potassium atom K41 is surrounded by six different mol­ecules. The six K⋯O inter­actions participating in the distorted octa­hedral coordination are shown in turquoise, the two longer ones in yellow.

In the crystal packing, the potassium ion K41 inter­acts with six mol­ecules of which two occur in the carb­oxy­lic acid form (Fig. 4 ▸). The distorted octa­hedron around K41 shares on opposite sides two oxygen atoms [at one side O40 and O40^ii^, at the other side O20^i^ and O20^iv^; symmetry codes: (i) *x* + 1, *y*, *z*, (ii) −*x* + 2, −*y* + 2, −*z* + 1, (iv) −*x* + 1, −*y* + 1, −*z* + 1] with inversion-related octa­hedra (Figs. 3 ▸ and 5 ▸). This results in parallel chains of octa­hedra running in the [010] direction and situated in the (002) plane. The K⋯K distances in the chains are 4.8084 (15) (for K41⋯K41^ii^) and 4.8353 (14) Å [for K41⋯K41^v^; symmetry code: (v) −*x* + 2, −*y* + 1, −*z* + 1].

**Figure 6:**
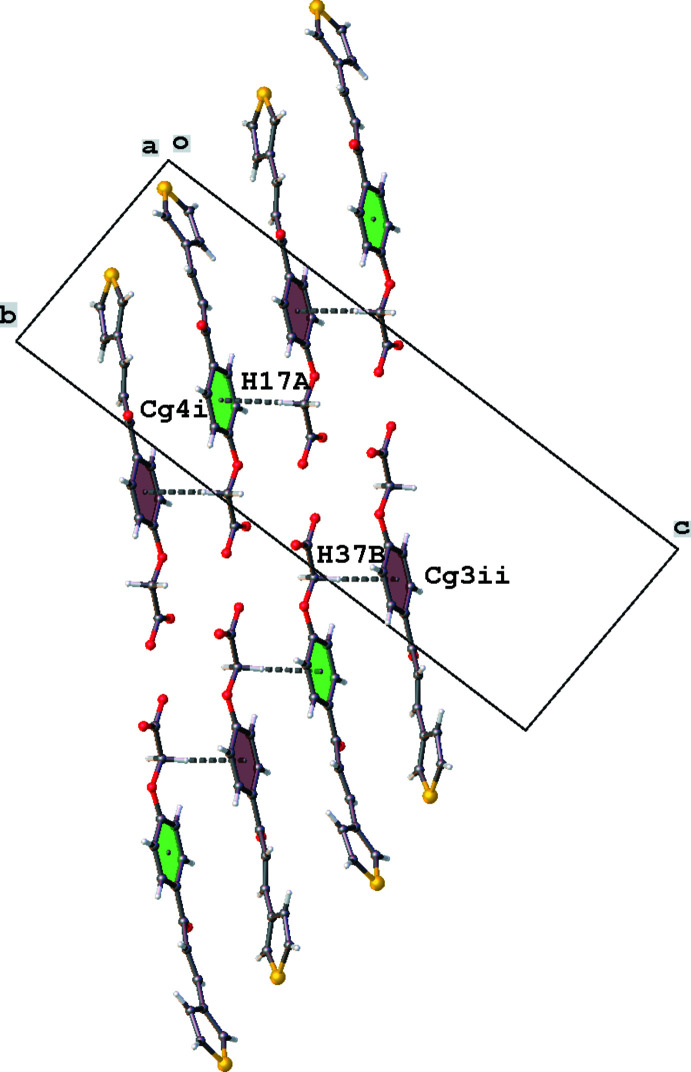
A view down the *a* axis of the inter­molecular C—H⋯π inter­actions of the title compound. Colour codes used: magenta for ring C10–C15, green for ring C30–C35. *Cg*3 and *Cg*4 are the centroids of the C10–C15 and C30–C35 rings, respectively. K atoms have been omitted.

Despite the presence of many aromatic rings, the crystal packing of the title compound does not show πany –π inter­actions. The shortest centroid–centroid distance is 4.735 (3) Å between thio­phene rings S1/C2–C5 and S21/C22–C25 with an angle between the rings of 52.3 (3)°. However, C—H⋯π inter­actions are present and give rise to a ladder-like chain also running in the [010] direction (Table 1 ▸, Fig. 6 ▸). In addition, neigbouring chains inter­act by short C28=O29⋯*Cg*1^v^ contacts [O29⋯*Cg*1^v^ = 3.652 (4) Å; *Cg*1 is the centroid of thio­phene ring S1/C2–C5; symmetry code: (v) −*x*, −*y* + 1, −*z* + 1].

The packing does not show any residual solvent-accessible voids.

## Hirshfeld surface analysis   

**Figure 7:**
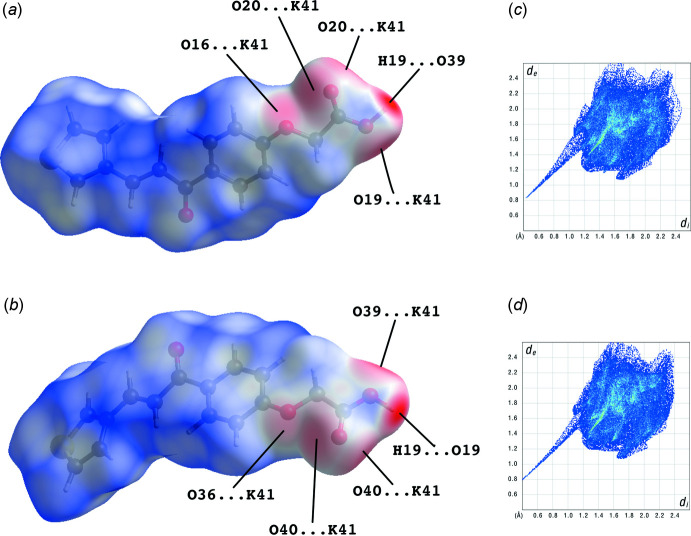
The Hirshfeld surfaces of mol­ecules (*a*) *A* and (*b*) *B* mapped over *d*_norm_ in the colour ranges −1.0475 to 1.0150 and −1.1101 to 1.0300 a.u., respectively, together with the full two-dimensional fingerprint plots for mol­ecules (*c*) *A* and (*d*) *B*.

The Hirshfeld surface analysis (Spackman & Jayatilaka, 2009 ▸) and the associated two-dimensional fingerprint plots (McKinnon *et al.*, 2007 ▸) were performed using *CrystalExplorer* (Turner *et al.*, 2017 ▸). The Hirshfeld surfaces of mol­ecules *A* and *B* mapped over *d*_norm_ are given in Fig. 7 ▸ *a* and *b*, respectively. The relative distributions from the different inter­atomic contacts to the Hirshfeld surfaces are presented in Table 2 ▸. The bright-red spots at atoms O19, H19 and O39 are indicative of the O19—H19⋯O39 hydrogen bond between the mol­ecules. The additional faint-red spots near atoms O16, O19, O20, O36, O39 and O40 concern the K⋯O inter­actions in the crystal structure. It should be noted that the Hirshfeld surfaces are almost identical for the two mol­ecules. The same is true for the fingerprint plots (Fig. 7 ▸ *c* and *d*). The sharp tips at *d*_e_ + *d*_i_ ≃ 1.4 Å arise from the O19—H19⋯O39 hydrogen bond. The principal contribution to the Hirshfeld surfaces involves H⋯H contacts at 31.6 and 31.9% for mol­ecules *A* and *B*, respectively. These are followed by C⋯H/H⋯C (21.1 and 20.0%) O⋯H/H⋯O (17.4 and 17.3%) and S⋯H/H⋯S (8.8 and 9.9%) contacts.

## Database survey   

**Figure 8:**
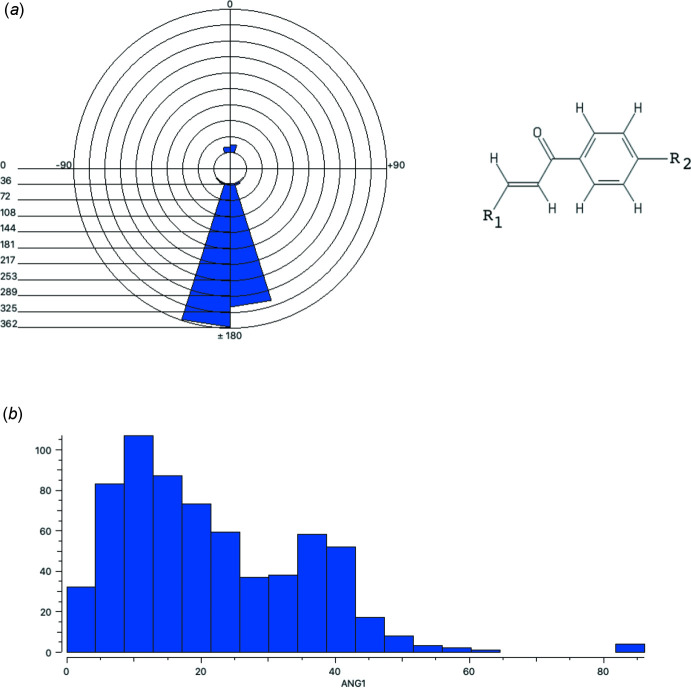
(*a*) Polar histogram of torsion angle *R*_1_—C=C—C. (*b*) Histogram of the dihedral angle between the planes of the double bond and the phenyl ring.

A search of the Cambridge Structural Database (CSD, Version 5.42, last update February 2021; Groom *et al.*, 2016 ▸) for the fragment *R*_1_—CH=CH—C(=O)—*p*-C_6_H_4_—*R*_2_ gave 619 hits (with C atoms double-bond acyclic). For only 33 cases (5.3%), the double bond has the *Z* configuration (Fig. 8 ▸ *a*). The histogram of the dihedral angle between the planes of the double bond and the phenyl ring shows values between 0.0 and 86.2° (Fig. 8 ▸ *b*). A search with thio­phene as *R*_1_ resulted in only four hits (CSD refcodes XOLJUG, XOLKAN, XOLKER and XOLKIV; Vu Quoc *et al.*, 2019*c* ▸) displaying the *E* configuration and dihedral angles in the range 6.7 to 15.8° (smaller than in the title compound). Only one structure was found for which *R*_2_ is the same as in the title compound (OCH_2_COOH; CSD refcode TAMJID; Abdul Ajees *et al.*, 2017 ▸). In this monohydrate, a water mol­ecule makes hydrogen bonds to the carboxyl groups of two neighbouring mol­ecules and in addition to a carbonyl of a third neighbouring enone moiety.

## Synthesis and crystallization   

**Figure 9:**
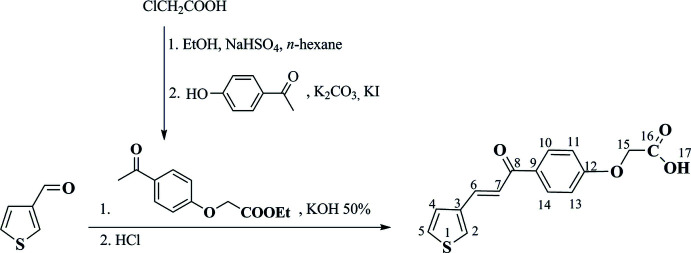
Reaction scheme for (*E*)-2-{4-[3-(thio­phen-3-yl)acrylo­yl]phen­oxy}acetic acid.

The synthetic pathway to synthesize the target compound, (*E*)-2-{4-[3-(thio­phen-3-yl)acrylo­yl]phen­oxy}acetic acid, is given in Fig. 9 ▸ (numbering on chemical formulas is only used for NMR spectroscopic analysis).

A mixture of ethyl 2-(4-acetyl­phen­oxy)acetate (5 mmol), 3-thio­phene­carbaldehyde (5 mmol) and 50 mL of ethanol was stirred in ice-cold water for 20 minutes. Then, 5 mL of 50% KOH solution was added dropwise to the reaction mixture, which was then stirred continuously for 5 h. At the end of the reaction, water was added to the reaction mixture and stirring was continued until all solids in the mixture were dissolved. Concentrated HCl was slowly added to the obtained solution until the solution changed from brown to yellow. The solution was then heated until crystals appeared. The solid then began to crystallize when the solution temperature started to decrease. The crystallized solid was filtered off, washed thoroughly with water and recrystallized from an ethanol–water mixture to give 2-{4-[3-(thio­phen-3-yl)acrylo­yl]phen­oxy}acetic acid (yield 62%) in the form of pale-yellow crystals (m.p. 455 K).

IR (Shimadzu FTIR-8400S, KBr, cm^−1^): 1017, 980 (=C—H bend), 1597 (C=C), 1659 (C=O), 3457 (*broad*, OH).

^1^H NMR [Bruker XL-500, 500 MHz, *d*_6_-DMSO, (ppm), *J* (Hz)]: 7.60 (*d*, 1H, H^2^), 7.42 (*m*, 1H, ^5^ *J* = 5.0, H^4^), 7.38 (*t*, 1H, ^4^ *J* = 5.0, H^5^), 7.81 (*d*, 1H, ^7^ *J* = 15.5, H^6^), 7.34 (*d*, 1H, ^6^ *J* = 15.5, H^7^), 8.03 (*t*, 2H, *J* = 9.0, H^10^ and H^14^), 7.02 (*m*, 2H, H^11^ and H^13^), 4.77 (*s*, 2H, H^15^).

^13^C NMR [Bruker XL-500, 125 MHz, *d*_6_-DMSO, (ppm)]: 121.81 (C2), 128.75 (C3), 127.01 (C4), 125.41 (C5), 132.67 (C6), 130.87 (C7), 171.85 (C8), 169.73 (C9), 138.39 (C10 and C14), 137.96 (C11 and C13), 114.65 (C12), 64.68 (C15), 189.09 (C16). Calculation for C_15_H_11_O_4_S: *M* = 287 au.

## Refinement   

Crystal data, data collection and structure refinement details are summarized in Table 3 ▸. Hydrogen atom H19 was located from a difference electron-density map and refined freely with an *U*_iso_(H) value of 1.5*U*_eq_ of the parent atom O19. The other H atoms were placed in idealized positions and included as riding contributions with an *U*_iso_(H) values of 1.2*U*_eq_ of the parent atom, with C—H distances of 0.93 (aromatic) and 0.97 Å (CH_2_). In the final cycles of refinement, 12 outliers with |error/e.s.d.| > 5.0 were omitted.

## Supplementary Material

Crystal structure: contains datablock(s) I. DOI: 10.1107/S2056989021004801/ey2007sup1.cif

Structure factors: contains datablock(s) I. DOI: 10.1107/S2056989021004801/ey2007Isup2.hkl

Supporting information file. DOI: 10.1107/S2056989021004801/ey2007Isup3.cml

CCDC reference: 2082049

Additional supporting information:  crystallographic information; 3D view; checkCIF report

## Figures and Tables

**Figure 5:**
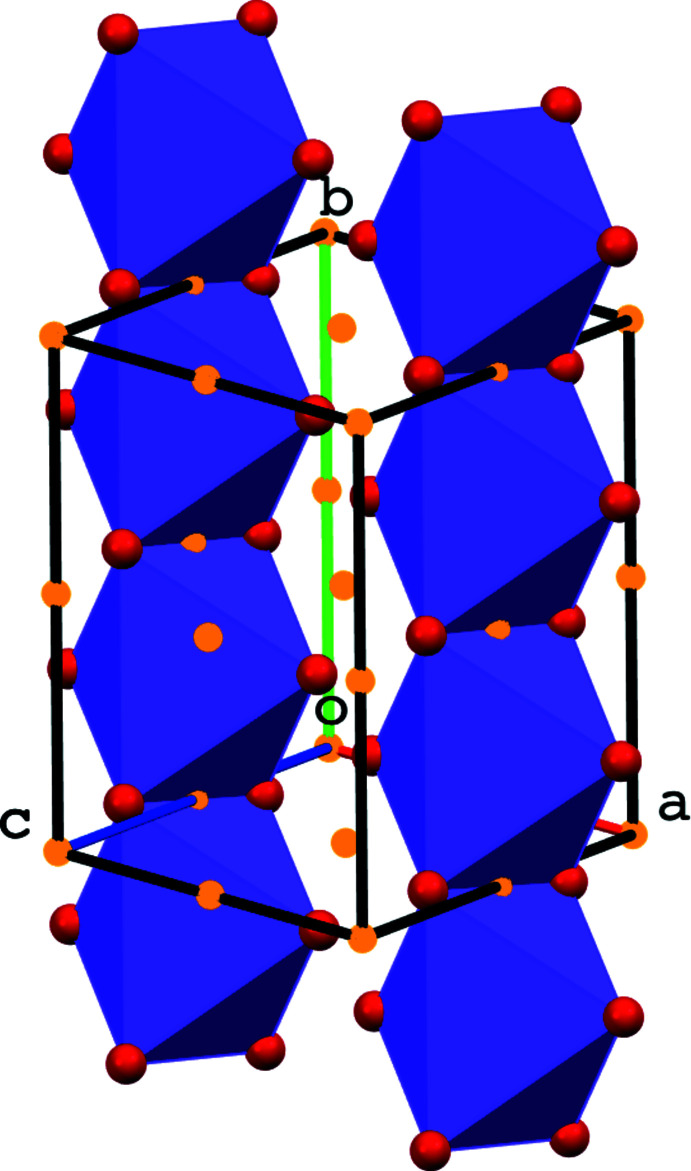
Parallel chains of K—O octa­hedra running in the [010] direction. Inversion centers are shown as yellow spheres.

**Table 1:** Hydrogen-bond geometry (Å, °) *Cg*3 and *Cg*4 are the centroids of rings C10–C15 and C30–C35, respectively.

*D*—H⋯*A*	*D*—H	H⋯*A*	*D*⋯*A*	*D*—H⋯*A*
O19—H19⋯O39	1.19 (4)	1.27 (4)	2.463 (3)	174 (4)
O19—H19⋯O40	1.19 (4)	2.47 (4)	3.259 (3)	122 (2)
C6—H6⋯O9	0.93	2.52	2.834 (6)	100
C26—H26⋯O29	0.93	2.49	2.814 (6)	100
C17—H17*A*⋯*Cg*4^i^	0.97	2.76	3.509 (4)	134
C37—H37*B*⋯*Cg*3^ii^	0.97	2.82	3.535 (4)	131

Symmetry codes: (i) -x+1, -y+2, -z+1; (ii) -x+1, -y+1, -z+1.

**Table 2:** Percentage contributions of inter­atomic contacts to the Hirshfeld surfaces of the two mol­ecules Mol­ecule *A* includes atoms S1–O20, mol­ecule *B* atoms S21–O40.

Contact	Mol­ecule *A*	Mol­ecule *B*
H⋯H	31.6	31.9
C⋯H/H⋯C	21.1	20.0
O⋯H/H⋯O	17.4	17.3
S⋯H/H⋯S	8.8	9.9
O⋯C/C⋯O	5.8	5.5
K⋯O/O⋯K	4.8	4.7
C⋯C	4.9	4.8
S⋯C/C⋯S	2.0	2.3
S⋯S	0.9	1.0
K⋯H/H⋯K	0.7	0.6

**Table 3:** Experimental details

Crystal data	
Chemical formula	[K(C_15_H_11_O_4_S)(C_15_H_12_O_4_S)]
*M*_r_	614.70
Crystal system, space group	Triclinic, *P*\overline{1}
Temperature (K)	293
*a*, *b*, *c* (Å)	6.0036 (3), 9.6432 (5), 25.0966 (16)
α, β, γ (°)	92.412 (4), 90.548 (4), 105.808 (4)
*V* (Å^3^)	1396.42 (14)
*Z*	2
Radiation type	Mo *K*α
μ (mm^−1^)	0.39
Crystal size (mm)	0.4 × 0.2 × 0.05

Data collection	
Diffractometer	Rigaku Oxford Diffraction SuperNova, Single source at offset/far, Eos
Absorption correction	Multi-scan (*CrysAlis PRO*; Rigaku OD, 2018 ▸)
*T*_min_, *T*_max_	0.863, 1.000
No. of measured, independent and observed [*I* > 2σ(*I*)] reflections	15169, 4742, 2836
*R*_int_	0.037
(sin θ/λ)_max_ (Å^−1^)	0.588

Refinement	
*R*[*F* ^2^ > 2σ(*F* ^2^)], *wR*(*F* ^2^), *S*	0.068, 0.211, 1.02
No. of reflections	4742
No. of parameters	373
H-atom treatment	H atoms treated by a mixture of independent and constrained refinement
Δρ_max_, Δρ_min_ (e Å^−3^)	0.59, −0.47

Computer programs: *CrysAlis PRO* (Rigaku OD, 2018 ▸), *SHELXT* (Sheldrick, 2015*a* ▸), *SHELXL2016/4* (Sheldrick, 2015*b* ▸) and *OLEX2* (Dolomanov *et al.*, 2009 ▸).

## supplementary crystallographic information

### Crystal data

**Table:**

[K(C_15_H_11_O_4_S)(C_15_H_12_O_4_S)]	*Z* = 2
*M**_r_* = 614.70	*F*(000) = 636
Triclinic, *P*1	*D*_x_ = 1.462 Mg m^−^^3^
*a* = 6.0036 (3) Å	Mo *K*α radiation, λ = 0.71073 Å
*b* = 9.6432 (5) Å	Cell parameters from 3156 reflections
*c* = 25.0966 (16) Å	θ = 3.2–24.6°
α = 92.412 (4)°	µ = 0.39 mm^−^^1^
β = 90.548 (4)°	*T* = 293 K
γ = 105.808 (4)°	Plate, colourless
*V* = 1396.42 (14) Å^3^	0.4 × 0.2 × 0.05 mm

### Data collection

**Table:**

Rigaku Oxford Diffraction SuperNova, Single source at offset/far, Eos diffractometer	4742 independent reflections
Radiation source: micro-focus sealed X-ray tube, SuperNova (Mo) X-ray Source	2836 reflections with *I* > 2σ(*I*)
Mirror monochromator	*R*_int_ = 0.037
Detector resolution: 15.9631 pixels mm^-1^	θ_max_ = 24.7°, θ_min_ = 2.4°
ω scans	*h* = −7→7
Absorption correction: multi-scan (CrysAlisPro; Rigaku OD, 2018)	*k* = −11→11
*T*_min_ = 0.863, *T*_max_ = 1.000	*l* = −29→29
15169 measured reflections	

### Refinement

**Table:**

Refinement on *F*^2^	Primary atom site location: dual
Least-squares matrix: full	Hydrogen site location: mixed
*R*[*F*^2^ > 2σ(*F*^2^)] = 0.068	H atoms treated by a mixture of independent and constrained refinement
*wR*(*F*^2^) = 0.211	*w* = 1/[σ^2^(*F*_o_^2^) + (0.111*P*)^2^ + 0.2689*P*] where *P* = (*F*_o_^2^ + 2*F*_c_^2^)/3
*S* = 1.02	(Δ/σ)_max_ < 0.001
4742 reflections	Δρ_max_ = 0.59 e Å^−^^3^
373 parameters	Δρ_min_ = −0.47 e Å^−^^3^
0 restraints	

### Special details

**Table:**

Geometry. All esds (except the esd in the dihedral angle between two l.s. planes) are estimated using the full covariance matrix. The cell esds are taken into account individually in the estimation of esds in distances, angles and torsion angles; correlations between esds in cell parameters are only used when they are defined by crystal symmetry. An approximate (isotropic) treatment of cell esds is used for estimating esds involving l.s. planes.

### Fractional atomic coordinates and isotropic or equivalent isotropic displacement parameters (Å^2^)

**Table:**

	*x*	*y*	*z*	*U*_iso_*/*U*_eq_	
S1	−0.2860 (3)	−0.46353 (17)	0.04899 (7)	0.0970 (6)	
C2	−0.0473 (9)	−0.4037 (5)	0.08824 (19)	0.0737 (15)	
H2	0.067798	−0.451127	0.090654	0.088*	
C3	−0.0407 (8)	−0.2770 (5)	0.11636 (17)	0.0590 (13)	
C4	−0.2427 (9)	−0.2331 (6)	0.1050 (2)	0.0793 (16)	
H4	−0.271387	−0.150647	0.120601	0.095*	
C5	−0.3963 (9)	−0.3270 (6)	0.06777 (19)	0.0773 (16)	
H5	−0.536358	−0.315058	0.055580	0.093*	
C6	0.1494 (8)	−0.2019 (5)	0.15232 (17)	0.0590 (13)	
H6	0.278677	−0.237405	0.152768	0.071*	
C7	0.1570 (8)	−0.0887 (5)	0.18434 (16)	0.0564 (12)	
H7	0.028240	−0.052797	0.186141	0.068*	
C8	0.3652 (8)	−0.0173 (4)	0.21754 (16)	0.0492 (11)	
O9	0.5535 (6)	−0.0384 (3)	0.20858 (12)	0.0645 (9)	
C10	0.3407 (7)	0.0826 (4)	0.26258 (15)	0.0415 (10)	
C11	0.1313 (7)	0.0763 (4)	0.28700 (16)	0.0481 (11)	
H11	−0.003355	0.010363	0.273775	0.058*	
C12	0.1206 (7)	0.1658 (4)	0.33031 (16)	0.0453 (10)	
H12	−0.019503	0.157608	0.347103	0.054*	
C13	0.3197 (6)	0.2689 (4)	0.34915 (14)	0.0345 (9)	
C14	0.5284 (6)	0.2788 (4)	0.32503 (15)	0.0399 (9)	
H14	0.661673	0.347244	0.337605	0.048*	
C15	0.5386 (7)	0.1867 (4)	0.28211 (15)	0.0424 (10)	
H15	0.679617	0.193833	0.265864	0.051*	
O16	0.2876 (4)	0.3529 (2)	0.39231 (10)	0.0382 (6)	
C17	0.4768 (6)	0.4721 (4)	0.40807 (14)	0.0319 (8)	
H17A	0.535908	0.527277	0.377332	0.038*	
H17B	0.600512	0.438362	0.423326	0.038*	
C18	0.3947 (6)	0.5661 (4)	0.44896 (14)	0.0306 (8)	
O19	0.5596 (4)	0.6761 (2)	0.46588 (10)	0.0377 (6)	
H19	0.510 (5)	0.754 (3)	0.4992 (13)	0.057*	
O20	0.1957 (4)	0.5391 (3)	0.46318 (11)	0.0438 (7)	
K41	0.99969 (12)	0.75075 (8)	0.50093 (4)	0.0469 (3)	
S21	1.2169 (3)	1.89226 (19)	0.97523 (6)	0.1039 (7)	
C22	0.9574 (9)	1.8038 (5)	0.9458 (2)	0.0810 (16)	
H22	0.815783	1.786740	0.962611	0.097*	
C23	0.9853 (9)	1.7605 (5)	0.89334 (18)	0.0594 (13)	
C24	1.2196 (10)	1.8010 (6)	0.8802 (2)	0.0792 (16)	
H24	1.273298	1.780776	0.846982	0.095*	
C25	1.3664 (9)	1.8759 (5)	0.9226 (2)	0.0764 (16)	
H25	1.526468	1.910936	0.920654	0.092*	
C26	0.7955 (8)	1.6804 (5)	0.85850 (17)	0.0609 (13)	
H26	0.647616	1.682595	0.868474	0.073*	
C27	0.8094 (8)	1.6051 (4)	0.81437 (16)	0.0544 (12)	
H27	0.955220	1.604702	0.802281	0.065*	
C28	0.6044 (8)	1.5216 (4)	0.78335 (16)	0.0507 (11)	
O29	0.4111 (6)	1.5305 (4)	0.79430 (13)	0.0712 (10)	
C30	0.6352 (7)	1.4222 (4)	0.73870 (15)	0.0417 (10)	
C31	0.8468 (7)	1.4318 (4)	0.71402 (16)	0.0511 (11)	
H31	0.978632	1.501336	0.726665	0.061*	
C32	0.8639 (7)	1.3410 (4)	0.67160 (16)	0.0471 (11)	
H32	1.005424	1.351138	0.655226	0.057*	
C33	0.6704 (6)	1.2342 (4)	0.65314 (15)	0.0362 (9)	
C34	0.4595 (7)	1.2207 (4)	0.67660 (15)	0.0415 (10)	
H34	0.329159	1.149650	0.664132	0.050*	
C35	0.4435 (7)	1.3150 (4)	0.71934 (15)	0.0437 (10)	
H35	0.301130	1.305726	0.735190	0.052*	
O36	0.7068 (4)	1.1507 (2)	0.61025 (10)	0.0382 (6)	
C37	0.5203 (6)	1.0305 (4)	0.59364 (14)	0.0337 (9)	
H37A	0.396265	1.063516	0.578152	0.040*	
H37B	0.460285	0.974311	0.624094	0.040*	
C38	0.6055 (6)	0.9381 (4)	0.55283 (13)	0.0294 (8)	
O39	0.4427 (4)	0.8279 (2)	0.53558 (10)	0.0377 (6)	
O40	0.8052 (4)	0.9662 (3)	0.53874 (11)	0.0437 (7)	

### Atomic displacement parameters (Å^2^)

**Table:**

	*U*^11^	*U*^22^	*U*^33^	*U*^12^	*U*^13^	*U*^23^
S1	0.1100 (13)	0.0799 (10)	0.0923 (11)	0.0172 (9)	−0.0278 (10)	−0.0307 (9)
C2	0.096 (4)	0.058 (3)	0.065 (3)	0.021 (3)	−0.022 (3)	−0.016 (3)
C3	0.075 (3)	0.054 (3)	0.048 (3)	0.019 (3)	−0.006 (2)	−0.009 (2)
C4	0.085 (4)	0.082 (4)	0.074 (3)	0.033 (3)	−0.012 (3)	−0.030 (3)
C5	0.071 (3)	0.104 (4)	0.062 (3)	0.035 (3)	−0.012 (3)	−0.020 (3)
C6	0.071 (3)	0.056 (3)	0.051 (3)	0.022 (2)	−0.009 (2)	−0.018 (2)
C7	0.064 (3)	0.059 (3)	0.047 (2)	0.021 (2)	−0.005 (2)	−0.011 (2)
C8	0.066 (3)	0.043 (2)	0.040 (2)	0.017 (2)	0.000 (2)	−0.007 (2)
O9	0.067 (2)	0.070 (2)	0.0599 (19)	0.0291 (18)	0.0033 (17)	−0.0232 (17)
C10	0.051 (3)	0.033 (2)	0.040 (2)	0.0112 (19)	−0.0026 (19)	−0.0040 (18)
C11	0.043 (2)	0.046 (2)	0.048 (2)	0.0037 (19)	−0.005 (2)	−0.017 (2)
C12	0.038 (2)	0.045 (2)	0.049 (2)	0.0065 (19)	0.0049 (19)	−0.007 (2)
C13	0.041 (2)	0.0283 (19)	0.035 (2)	0.0125 (17)	−0.0021 (18)	−0.0055 (17)
C14	0.040 (2)	0.033 (2)	0.044 (2)	0.0079 (18)	−0.0013 (19)	−0.0081 (18)
C15	0.042 (2)	0.045 (2)	0.043 (2)	0.0168 (19)	0.0027 (19)	−0.0065 (19)
O16	0.0329 (14)	0.0307 (13)	0.0467 (15)	0.0037 (11)	0.0042 (12)	−0.0143 (12)
C17	0.0271 (18)	0.0277 (18)	0.041 (2)	0.0080 (15)	0.0047 (16)	−0.0023 (17)
C18	0.034 (2)	0.0231 (18)	0.036 (2)	0.0108 (16)	0.0022 (17)	0.0031 (16)
O19	0.0281 (13)	0.0313 (13)	0.0503 (16)	0.0046 (11)	−0.0025 (12)	−0.0134 (12)
O20	0.0313 (15)	0.0346 (15)	0.0600 (17)	0.0013 (12)	0.0132 (13)	−0.0118 (13)
K41	0.0229 (5)	0.0337 (5)	0.0827 (7)	0.0078 (4)	−0.0027 (4)	−0.0122 (5)
S21	0.1230 (14)	0.1059 (13)	0.0789 (11)	0.0321 (11)	−0.0247 (10)	−0.0399 (10)
C22	0.087 (4)	0.082 (4)	0.067 (3)	0.017 (3)	−0.003 (3)	−0.030 (3)
C23	0.076 (3)	0.053 (3)	0.050 (3)	0.021 (3)	−0.006 (3)	−0.014 (2)
C24	0.086 (4)	0.081 (4)	0.061 (3)	0.011 (3)	0.000 (3)	−0.021 (3)
C25	0.062 (3)	0.068 (3)	0.089 (4)	0.006 (3)	−0.003 (3)	−0.024 (3)
C26	0.073 (3)	0.057 (3)	0.056 (3)	0.024 (3)	−0.002 (3)	−0.010 (2)
C27	0.067 (3)	0.053 (3)	0.043 (2)	0.018 (2)	−0.002 (2)	−0.011 (2)
C28	0.066 (3)	0.043 (2)	0.043 (2)	0.017 (2)	0.001 (2)	−0.002 (2)
O29	0.068 (2)	0.078 (2)	0.071 (2)	0.0307 (19)	0.0031 (18)	−0.0287 (18)
C30	0.053 (3)	0.038 (2)	0.036 (2)	0.018 (2)	0.0015 (19)	−0.0054 (18)
C31	0.045 (2)	0.048 (2)	0.055 (3)	0.007 (2)	−0.009 (2)	−0.020 (2)
C32	0.039 (2)	0.044 (2)	0.054 (3)	0.0077 (19)	0.000 (2)	−0.016 (2)
C33	0.040 (2)	0.0291 (19)	0.041 (2)	0.0136 (17)	0.0015 (18)	−0.0043 (17)
C34	0.042 (2)	0.031 (2)	0.048 (2)	0.0050 (17)	0.0015 (19)	−0.0088 (19)
C35	0.042 (2)	0.046 (2)	0.043 (2)	0.0130 (19)	0.0056 (19)	0.000 (2)
O36	0.0343 (14)	0.0296 (13)	0.0467 (15)	0.0042 (11)	0.0040 (12)	−0.0128 (12)
C37	0.0314 (19)	0.0255 (18)	0.043 (2)	0.0070 (16)	0.0019 (17)	−0.0045 (17)
C38	0.030 (2)	0.0262 (18)	0.034 (2)	0.0111 (16)	−0.0027 (17)	−0.0014 (16)
O39	0.0256 (13)	0.0333 (13)	0.0511 (16)	0.0053 (11)	−0.0033 (12)	−0.0141 (12)
O40	0.0317 (15)	0.0330 (14)	0.0632 (18)	0.0042 (12)	0.0130 (13)	−0.0073 (13)

### Geometric parameters (Å, º)

**Table:**

S1—C2	1.682 (5)	K41—K41^iv^	4.8084 (15)
S1—C5	1.680 (5)	K41—K41^v^	4.8353 (14)
C2—H2	0.9300	K41—O36^iv^	3.347 (3)
C2—C3	1.375 (6)	K41—O39^iii^	2.685 (2)
C3—C4	1.419 (6)	K41—O40^iv^	2.885 (3)
C3—C6	1.455 (6)	K41—O40	2.785 (2)
C4—H4	0.9300	S21—C22	1.703 (5)
C4—C5	1.415 (6)	S21—C25	1.631 (5)
C5—H5	0.9300	C22—H22	0.9300
C6—H6	0.9300	C22—C23	1.390 (6)
C6—C7	1.318 (5)	C23—C24	1.400 (7)
C7—H7	0.9300	C23—C26	1.451 (6)
C7—C8	1.481 (6)	C24—H24	0.9300
C8—O9	1.223 (5)	C24—C25	1.413 (6)
C8—C10	1.489 (5)	C25—H25	0.9300
C10—C11	1.391 (5)	C26—H26	0.9300
C10—C15	1.399 (5)	C26—C27	1.314 (5)
C11—H11	0.9300	C27—H27	0.9300
C11—C12	1.372 (5)	C27—C28	1.469 (6)
C12—H12	0.9300	C28—O29	1.220 (5)
C12—C13	1.394 (5)	C28—C30	1.490 (5)
C13—C14	1.378 (5)	C30—C31	1.400 (6)
C13—O16	1.372 (4)	C30—C35	1.388 (5)
C14—H14	0.9300	C31—H31	0.9300
C14—C15	1.380 (5)	C31—C32	1.372 (5)
C15—H15	0.9300	C32—H32	0.9300
O16—C17	1.417 (4)	C32—C33	1.387 (5)
O16—K41^i^	3.245 (3)	C33—C34	1.377 (5)
C17—H17A	0.9700	C33—O36	1.372 (4)
C17—H17B	0.9700	C34—H34	0.9300
C17—C18	1.514 (5)	C34—C35	1.398 (5)
C18—O19	1.292 (4)	C35—H35	0.9300
C18—O20	1.212 (4)	O36—K41^iv^	3.347 (3)
O19—H19	1.19 (3)	O36—C37	1.420 (4)
O19—K41	2.672 (2)	C37—H37A	0.9700
O20—K41^ii^	2.760 (2)	C37—H37B	0.9700
O20—K41^i^	2.906 (3)	C37—C38	1.512 (5)
K41—O16^i^	3.245 (3)	C38—O39	1.288 (4)
K41—O19	2.672 (2)	C38—O40	1.215 (4)
K41—H19	2.95 (3)	O39—H19	1.27 (3)
K41—O20^i^	2.906 (3)	O39—K41^ii^	2.685 (2)
K41—O20^iii^	2.760 (2)	O40—K41^iv^	2.885 (3)
			
C5—S1—C2	94.1 (2)	K41^iv^—K41—H19	73.6 (6)
S1—C2—H2	123.6	K41^iv^—K41—K41^v^	178.88 (4)
C3—C2—S1	112.8 (4)	O36^iv^—K41—H19	116.2 (6)
C3—C2—H2	123.6	O36^iv^—K41—K41^v^	99.62 (4)
C2—C3—C4	110.3 (4)	O36^iv^—K41—K41^iv^	79.38 (5)
C2—C3—C6	123.5 (4)	O39^iii^—K41—O16^i^	103.47 (7)
C4—C3—C6	126.2 (4)	O39^iii^—K41—H19	156.1 (7)
C3—C4—H4	123.2	O39^iii^—K41—O20^i^	105.57 (8)
C5—C4—C3	113.5 (4)	O39^iii^—K41—O20^iii^	73.16 (7)
C5—C4—H4	123.2	O39^iii^—K41—K41^v^	89.77 (5)
S1—C5—H5	125.3	O39^iii^—K41—K41^iv^	90.48 (5)
C4—C5—S1	109.3 (4)	O39^iii^—K41—O36^iv^	76.94 (7)
C4—C5—H5	125.3	O39^iii^—K41—O40	106.49 (8)
C3—C6—H6	116.7	O39^iii^—K41—O40^iv^	74.93 (7)
C7—C6—C3	126.6 (4)	O40—K41—O16^i^	70.70 (7)
C7—C6—H6	116.7	O40^iv^—K41—O16^i^	131.58 (7)
C6—C7—H7	119.1	O40^iv^—K41—H19	97.8 (6)
C6—C7—C8	121.7 (4)	O40—K41—H19	51.1 (6)
C8—C7—H7	119.1	O40^iv^—K41—O20^i^	177.80 (7)
C7—C8—C10	118.2 (4)	O40—K41—O20^i^	117.64 (8)
O9—C8—C7	121.6 (4)	O40^iv^—K41—K41^v^	147.81 (6)
O9—C8—C10	120.2 (4)	O40—K41—K41^v^	148.16 (6)
C11—C10—C8	123.3 (4)	O40—K41—K41^iv^	32.63 (6)
C11—C10—C15	118.1 (3)	O40^iv^—K41—K41^iv^	31.38 (5)
C15—C10—C8	118.5 (4)	O40^iv^—K41—O36^iv^	49.80 (6)
C10—C11—H11	119.5	O40—K41—O36^iv^	110.51 (7)
C12—C11—C10	121.0 (4)	O40—K41—O40^iv^	64.01 (9)
C12—C11—H11	119.5	C25—S21—C22	94.4 (2)
C11—C12—H12	120.0	S21—C22—H22	124.5
C11—C12—C13	120.0 (4)	C23—C22—S21	111.1 (4)
C13—C12—H12	120.0	C23—C22—H22	124.5
C14—C13—C12	120.0 (3)	C22—C23—C24	110.5 (4)
O16—C13—C12	114.8 (3)	C22—C23—C26	123.7 (5)
O16—C13—C14	125.2 (3)	C24—C23—C26	125.8 (4)
C13—C14—H14	120.2	C23—C24—H24	123.3
C13—C14—C15	119.6 (3)	C23—C24—C25	113.4 (5)
C15—C14—H14	120.2	C25—C24—H24	123.3
C10—C15—H15	119.4	S21—C25—H25	124.6
C14—C15—C10	121.2 (3)	C24—C25—S21	110.7 (4)
C14—C15—H15	119.4	C24—C25—H25	124.6
C13—O16—C17	116.7 (3)	C23—C26—H26	116.4
C13—O16—K41^i^	127.8 (2)	C27—C26—C23	127.1 (5)
C17—O16—K41^i^	106.87 (18)	C27—C26—H26	116.4
O16—C17—H17A	110.0	C26—C27—H27	118.6
O16—C17—H17B	110.0	C26—C27—C28	122.8 (4)
O16—C17—C18	108.7 (3)	C28—C27—H27	118.6
H17A—C17—H17B	108.3	C27—C28—C30	118.8 (4)
C18—C17—H17A	110.0	O29—C28—C27	121.1 (4)
C18—C17—H17B	110.0	O29—C28—C30	120.1 (4)
O19—C18—C17	112.2 (3)	C31—C30—C28	123.8 (4)
O20—C18—C17	122.6 (3)	C35—C30—C28	118.7 (4)
O20—C18—O19	125.2 (3)	C35—C30—C31	117.5 (3)
C18—O19—H19	116.3 (15)	C30—C31—H31	119.3
C18—O19—K41	142.1 (2)	C32—C31—C30	121.5 (4)
K41—O19—H19	91.2 (14)	C32—C31—H31	119.3
C18—O20—K41^ii^	122.3 (2)	C31—C32—H32	120.0
C18—O20—K41^i^	115.7 (2)	C31—C32—C33	120.0 (4)
K41^ii^—O20—K41^i^	117.15 (9)	C33—C32—H32	120.0
O16^i^—K41—H19	63.9 (6)	C34—C33—C32	120.2 (3)
O16^i^—K41—K41^v^	79.07 (5)	O36—C33—C32	115.1 (3)
O16^i^—K41—K41^iv^	101.93 (5)	O36—C33—C34	124.7 (3)
O16^i^—K41—O36^iv^	178.61 (5)	C33—C34—H34	120.4
O19—K41—O16^i^	76.94 (7)	C33—C34—C35	119.3 (3)
O19—K41—H19	23.9 (6)	C35—C34—H34	120.4
O19—K41—O20^i^	74.97 (7)	C30—C35—C34	121.6 (4)
O19—K41—O20^iii^	107.07 (8)	C30—C35—H35	119.2
O19—K41—K41^v^	90.67 (5)	C34—C35—H35	119.2
O19—K41—K41^iv^	89.07 (5)	C33—O36—K41^iv^	129.6 (2)
O19—K41—O36^iv^	102.66 (7)	C33—O36—C37	117.0 (3)
O19—K41—O39^iii^	179.45 (8)	C37—O36—K41^iv^	104.73 (18)
O19—K41—O40	73.28 (7)	O36—C37—H37A	109.9
O19—K41—O40^iv^	104.52 (8)	O36—C37—H37B	109.9
O20^i^—K41—O16^i^	50.51 (6)	O36—C37—C38	109.1 (3)
O20^iii^—K41—O16^i^	109.72 (7)	H37A—C37—H37B	108.3
O20^i^—K41—H19	82.6 (6)	C38—C37—H37A	109.9
O20^iii^—K41—H19	129.3 (6)	C38—C37—H37B	109.9
O20^iii^—K41—O20^i^	62.85 (9)	O39—C38—C37	112.1 (3)
O20^i^—K41—K41^iv^	150.25 (6)	O40—C38—C37	122.9 (3)
O20^i^—K41—K41^v^	30.52 (5)	O40—C38—O39	125.0 (3)
O20^iii^—K41—K41^v^	32.32 (6)	K41^ii^—O39—H19	94.4 (13)
O20^iii^—K41—K41^iv^	146.88 (7)	C38—O39—H19	112.4 (14)
O20^iii^—K41—O36^iv^	69.08 (7)	C38—O39—K41^ii^	142.1 (2)
O20^i^—K41—O36^iv^	128.11 (6)	K41—O40—K41^iv^	115.99 (9)
O20^iii^—K41—O40^iv^	115.51 (8)	C38—O40—K41^iv^	116.9 (2)
O20^iii^—K41—O40	179.49 (8)	C38—O40—K41	121.3 (2)
K41^v^—K41—H19	106.5 (6)		
			
S1—C2—C3—C4	1.2 (6)	K41^iv^—O36—C37—C38	39.0 (3)
S1—C2—C3—C6	−178.9 (4)	S21—C22—C23—C24	0.6 (6)
C2—S1—C5—C4	0.4 (5)	S21—C22—C23—C26	178.8 (4)
C2—C3—C4—C5	−0.9 (7)	C22—S21—C25—C24	0.0 (4)
C2—C3—C6—C7	−172.8 (5)	C22—C23—C24—C25	−0.6 (7)
C3—C4—C5—S1	0.3 (6)	C22—C23—C26—C27	−161.5 (5)
C3—C6—C7—C8	−177.2 (4)	C23—C24—C25—S21	0.3 (6)
C4—C3—C6—C7	7.1 (9)	C23—C26—C27—C28	176.8 (4)
C5—S1—C2—C3	−0.9 (4)	C24—C23—C26—C27	16.4 (8)
C6—C3—C4—C5	179.2 (5)	C25—S21—C22—C23	−0.4 (4)
C6—C7—C8—O9	16.3 (7)	C26—C23—C24—C25	−178.8 (4)
C6—C7—C8—C10	−163.9 (4)	C26—C27—C28—O29	7.8 (7)
C7—C8—C10—C11	23.2 (6)	C26—C27—C28—C30	−170.8 (4)
C7—C8—C10—C15	−158.0 (4)	C27—C28—C30—C31	−19.3 (6)
C8—C10—C11—C12	176.3 (4)	C27—C28—C30—C35	162.1 (4)
C8—C10—C15—C14	−177.6 (4)	C28—C30—C31—C32	−177.4 (4)
O9—C8—C10—C11	−157.1 (4)	C28—C30—C35—C34	178.3 (4)
O9—C8—C10—C15	21.7 (6)	O29—C28—C30—C31	162.1 (4)
C10—C11—C12—C13	2.6 (6)	O29—C28—C30—C35	−16.5 (6)
C11—C10—C15—C14	1.2 (6)	C30—C31—C32—C33	−1.7 (7)
C11—C12—C13—C14	−1.5 (6)	C31—C30—C35—C34	−0.5 (6)
C11—C12—C13—O16	179.5 (3)	C31—C32—C33—C34	1.3 (6)
C12—C13—C14—C15	0.3 (6)	C31—C32—C33—O36	179.4 (4)
C12—C13—O16—C17	−171.3 (3)	C32—C33—C34—C35	−0.5 (6)
C12—C13—O16—K41^i^	45.4 (4)	C32—C33—O36—K41^iv^	−44.3 (4)
C13—C14—C15—C10	−0.2 (6)	C32—C33—O36—C37	173.4 (3)
C13—O16—C17—C18	169.6 (3)	C33—C34—C35—C30	0.1 (6)
C14—C13—O16—C17	9.7 (5)	C33—O36—C37—C38	−170.2 (3)
C14—C13—O16—K41^i^	−133.6 (3)	C34—C33—O36—K41^iv^	133.7 (3)
C15—C10—C11—C12	−2.4 (6)	C34—C33—O36—C37	−8.6 (5)
O16—C13—C14—C15	179.2 (3)	C35—C30—C31—C32	1.3 (6)
O16—C17—C18—O19	178.9 (3)	O36—C33—C34—C35	−178.4 (3)
O16—C17—C18—O20	−1.5 (5)	O36—C37—C38—O39	−179.0 (3)
C17—C18—O19—K41	−46.1 (5)	O36—C37—C38—O40	1.2 (5)
C17—C18—O20—K41^i^	52.3 (4)	C37—C38—O39—K41^ii^	44.9 (5)
C17—C18—O20—K41^ii^	−153.2 (2)	C37—C38—O40—K41	153.2 (3)
O19—C18—O20—K41^ii^	26.3 (5)	C37—C38—O40—K41^iv^	−54.7 (4)
O19—C18—O20—K41^i^	−128.2 (3)	O39—C38—O40—K41	−26.5 (5)
O20—C18—O19—K41	134.3 (3)	O39—C38—O40—K41^iv^	125.6 (3)
K41^i^—O16—C17—C18	−40.0 (3)	O40—C38—O39—K41^ii^	−135.4 (3)

Symmetry codes: (i) −*x*+1, −*y*+1, −*z*+1; (ii) *x*−1, *y*, *z*; (iii) *x*+1, *y*, *z*; (iv) −*x*+2, −*y*+2, −*z*+1; (v) −*x*+2, −*y*+1, −*z*+1.

### Hydrogen-bond geometry (Å, º)

*Cg*3 and *Cg*4 are the centroids of rings C10–C15 and C30–C35, respectively.

**Table:**

*D*—H···*A*	*D*—H	H···*A*	*D*···*A*	*D*—H···*A*
O19—H19···O39	1.19 (4)	1.27 (4)	2.463 (3)	174 (4)
O19—H19···O40	1.19 (4)	2.47 (4)	3.259 (3)	122 (2)
C6—H6···O9	0.93	2.52	2.834 (6)	100
C26—H26···O29	0.93	2.49	2.814 (6)	100
C17—H17*A*···*Cg*4^vi^	0.97	2.76	3.509 (4)	134
C37—H37*B*···*Cg*3^i^	0.97	2.82	3.535 (4)	131

Symmetry codes: (i) −*x*+1, −*y*+1, −*z*+1; (vi) −*x*+1, −*y*+2, −*z*+1.
